# Fasciite nodulaire de la fosse infra-temporale: à propos d’un cas

**DOI:** 10.11604/pamj.2018.31.106.16636

**Published:** 2018-10-12

**Authors:** Abdelouahid Taleuan, Dounia Kamal, Moad Sebti, Moahamed Nourdine Elalami

**Affiliations:** 1 Service d’Oto-Rhino-Laryngologie et Chirurgie Cervico-Faciale, CHU Hassan II, Fès, Maroc

**Keywords:** Fasciite nodulaire, Fosse infra-temporale, Tumeur, Nodular fasciitis, infratemporal fossa, tumor

## Abstract

La fasciite nodulaire est une lésion bénigne à prolifération rapide de cellules myofibroblastiques, qui se développe aux dépens d'un fascia musculaire au sein du tissu sous-cutané. Sa croissance rapide et sa richesse cellulaire et mitotique font qu'elle est souvent prise à tort pour un sarcome. D'où l'importance de poser un diagnostic précis pour éviter des chirurgies inutiles et souvent mutilantes.Nous rapportons dans cette observation une localisation exceptionnelle de cette tumeur au niveau de la fosse infra-temporale.

## Abstract (en)

Nodular fasciitis is a benign lesion characterized by a rapidly growing proliferation of myofibroblastic cells which develop at the expense of a muscular fascia inside the subcutaneous tissue. It is often confused with sarcoma due to its rapid growth and its cellular and mitotic richness. Hence, the importance of accurate diagnosis to avoid unnecessary and often mutilating surgeries. This study presents a case of nodular fasciitis arising in an uncommon site, the infratemporal fossa.

## Introduction

La fasciite nodulaire (FN), également désigné fibrosite nodulaire, fibromatose sous cutanée, fasciite proliférante, fasciite pseudo-sarcomateuse, est la plus commune des proliférations fibroblastiques bénignes des parties molles d'étiologie précise encore inconnue, décrite pour la première fois en 1955 par Konwaler. Environ la moitié des cas déclarés se retrouvent au niveau des membres supérieurs et jusqu'à 20 % des lésions sont présentes dans la région cervicofacial [1]. L'importance de cette lésion tient à sa croissance rapide et à ses aspects morphologiques inquiétants mimant une tumeur maligne notamment un fibrosarcome. Nous rapportons dans cette observation une localisation rare de cette tumeur au niveau de la fosse infra-temporale (FIT), qui nous a posé un problème diagnostique clinique et radiologique.

## Patient et observation

**Figure 1:**
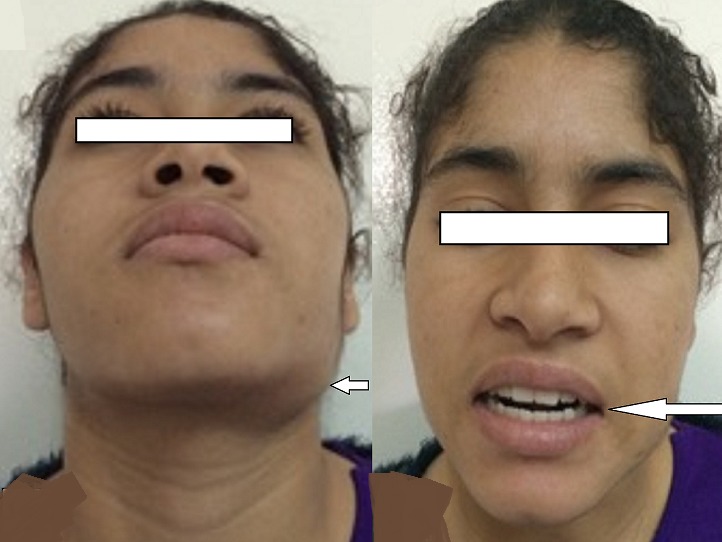
Photos préopératoires de la patiente: montrant une tuméfaction de l’angle mandibulaire gauche avec un trismus serré

**Figure 2:**
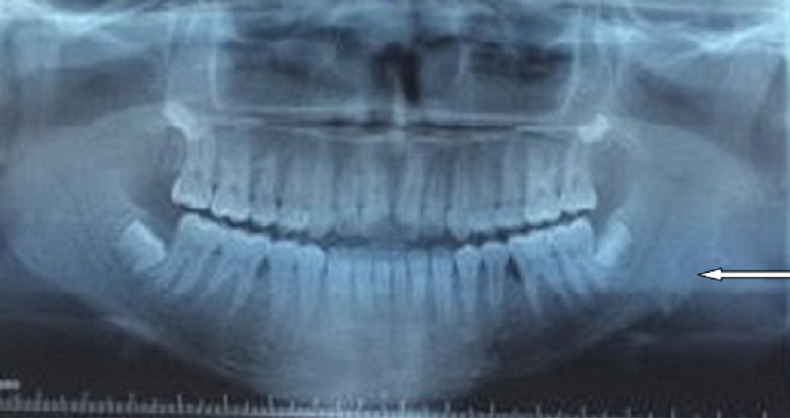
Panoramique dentaire montrant une lyse de l’angle mandibulaire gauche

**Figure 3:**
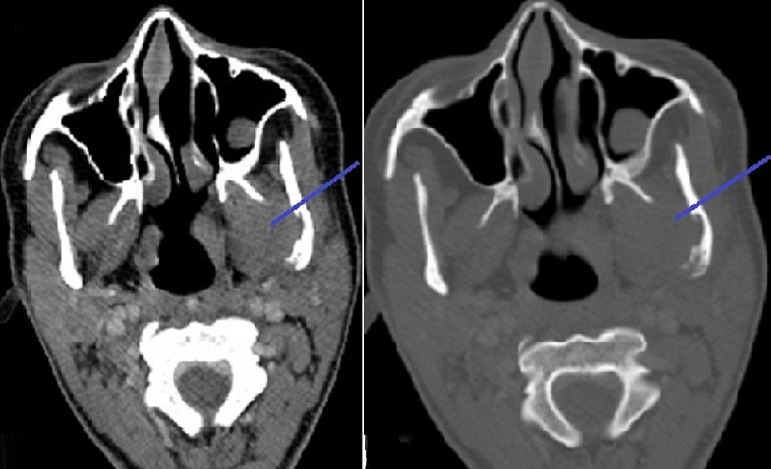
TDM en coupe axiale montrant une formation tissulaire de la FIT avec lyse de la branche montante de la mandibule à gauche

**Figure 4:**
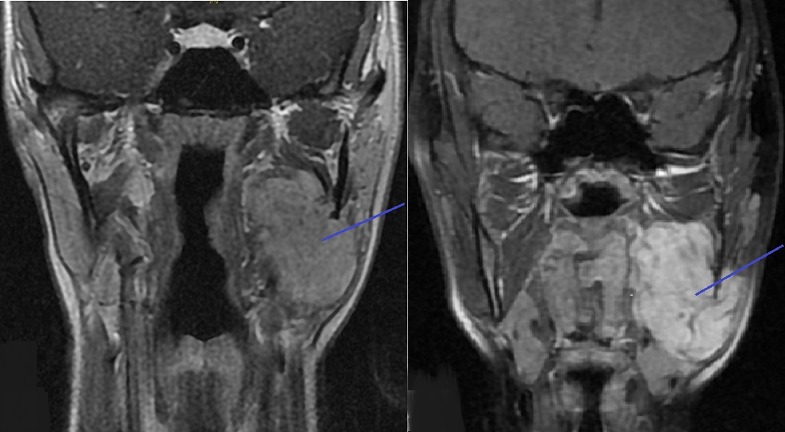
IRM en coupes coronales: montrant un processus lésionnel ptérygoïdien

**Figure 5:**
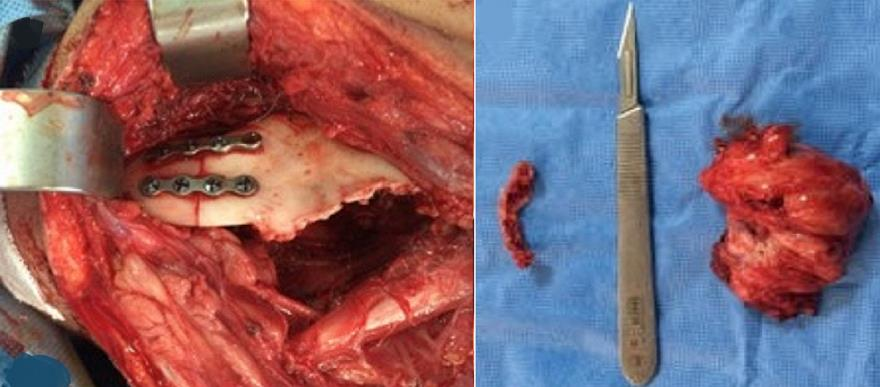
Photos per-opératoires: de la pièce opératoire et de la voie d’abord chirurgicale

**Figure 6:**
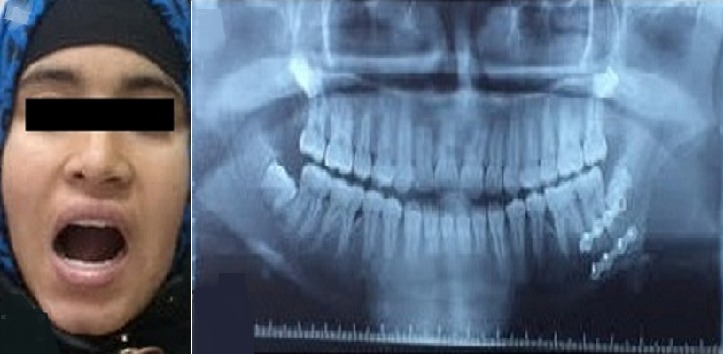
Photos postopératoires montrant nette amélioration du trismus

Il s'agit d'une jeune patiente âgée de 16 ans, lycéenne, sans antécédents pathologiques notables notamment pas de notion de traumatisme faciale, et qui a été adressée au CHU pour prise en charge d'une tuméfaction mandibulaire gauche évoluant depuis environ 2 ans, augmentant progressivement du volume (Figure 1). L'examen clinique a mis en évidence une tuméfaction de l'angle mandibulaire gauche, de trois cm de diamètre, indolore, dure, fixe au plan profond et ne s'accompagnant d'aucun signe inflammatoire local ni d'adénopathie satellite. Par ailleurs un trismus serré a été noté (Figure 1). Le bilan biologique inflammatoire était sans particularité. La radiographie panoramique dentaire a révélé une lyse osseuse de l'angle mandibulaire gauche (Figure 2). La tomodensitométrie (TDM) cervico-faciale a mis en évidence un processus tissulaire centré sur les muscles ptérygoïdiens gauches rehaussé après injection de produit de contraste (PC), avec une lyse osseuse partielle de l'angle mandibulaire gauche et de l'apophyse ptérygoïdienne gauche (Figure 3). En imagerie par résonnance magnétique (IRM) cervico-faciale il s'agissait d'un processus tissulaire mesurant 36mmx41mmx51mm, au niveau de la FIT mal limité de contours irréguliers, avec un hyposignal en T1, un hypersignal en T2 et rehaussé de façon hétérogène et intense après injection de PC. Cet aspect était évocateur d'un sarcome (Figure 4). La biopsie de la masse a fait l'objet d'un examen histopathologique qui a montré une prolifération de cellules allongées, disposées sur un fond lâche partiellement fibreux parcouru par de nombreux capillaires avec parfois une extravasation des globules rouges. On a complété par une étude immunohistochimique (IHC), qui était en faveur d'une fasciite nodulaire. Devant la confirmation diagnostique, une exérèse chirurgicale de la tumeur a été réalisée en monobloc grâce à une voie d'abord combinée, cervicale et trans-mandibulaire (Figure 5). Les suites opératoires ont été simples et l'évolution a été favorable, marquée par la disparition du trismus et l'absence de récidive avec un recul d'un an (Figure 6).

## Discussion

Parmi les tumeurs des tissus mous, la FN reste un processus tumoral bénin relativement fréquent dans les 30 premières années de vie [2]. Sa pathogénie est mal connue. Un traumatisme ou un processus inflammatoire local pourrait constituer un facteur déclenchant. Elle peut inquiéter par son évolution clinique rapide, son caractère infiltrant ou mal limité, et son aspect histologique sarcomatoïde. Mais c'est une lésion bénigne, sans pouvoir métastatique connu, et ne récidivant que très rarement [2]. La localisation cervico-faciale, peu fréquente (7% à 20%), est toutefois dominante chez l'enfant ou l'adolescent; Les sites les plus communément atteints sont la peau recouvrant l'arcade zygomatique et la partie antérieure et postérieure de la mandibule, la muqueuse buccale, la parotide, l'orbite et la nuque [1]. À notre connaissance, la localisation infra-temporale de la FN n'a encore jamais été rapportée. C'est une tumeur qui touche indifféremment l'homme et la femme et qui croît rapidement en quelques semaines [3]. Seulement 7 % des cas ont une lésion connue depuis plus d'un an; c'est le cas de notre patiente. L'aspect macroscopique n'est pas un recours valable pour établir le diagnostic, car la lésion peut être située dans les tissussous-cutanés, aponévrotiques ou intramusculaires .Les formes intramusculaires- Cas de notre patiente- sont plus volumineuses, plus profondes et moins bien limitées, de sorte que l'examen clinique et l'IRM peuvent évoquer un sarcome [4]. En IRM, les caractéristiques intrinsèques du signal reflètent le type histologique [4]. En effet, les lésions myxoïdes et cellulaires sont iso-intenses ou hyper-intenses par comparaison au muscle sur les images pondérées en T1 et iso-intenses ou hyper-intenses par comparaison au tissu adipeux sur les images pondérées en T2. Cet aspect peut évoquer un sarcome. Les lésions fibreuses, en revanche, sont nettement hypo-intenses sur les images echopondérées en T1 et T2. L'injection de gadolinium est habituellement suivie d'une nette prise de contraste [5]. En microscopie, les cellules fusiformes ont des mitoses fréquentes sans forme atypique. De nombreux vaisseaux sanguins radiés partent du centre hémorragique, réalisant un phénomène de zone expliquant un aspect richement vascularisé à l'échographie et le rehaussement de la tumeur par le PC à l'IRM. En IHC, les cellules fusiformes expriment l'actine du muscle lisse [6]. En raison de ses caractéristiques histologiques parfois alarmantes, certains pathologistes ont tendance à confondre la FN avec d'autres tumeurs à cellules fusiformes notamment: un Sarcome, un myxome, un histiocytome et une fibromatose [4]. L´excision chirurgicale conservatrice reste le traitement de choix de ces lésions bénignes [7]. La régression spontanée est largement signalée dans la littérature [3]. C'est pour cette raison, qu'une période de suivi de 4 à 6 semaines après biopsie devrait être envisagée en raison de la possibilité de régression spontanée de la tumeur. Si la résolution n'a pas eu lieu après cette période, une thérapie chirurgicale conservatrice devrait être effectuée [3]. Après résection les récidives sont extrêmement rares (dans 1 % à 2 % des cas) [2].

## Conclusion

Notre cas démontre que, bien que rare dans la FIT, la fasciite nodulaire doit être inclus dans le diagnostic différentiel d'une masse des tissus mou de cette région d'autant plus si elle est de développement récent et rapide. L'examen histologique doit être réalisé par des anatomopathologistes expérimentés pour éviter un diagnostic erroné de sarcome avec des conséquences potentiellement dramatiques.

## Conflits d’intérêts

Les auteurs ne déclarent aucun conflit d´intérêts.
